# Financing fragility and pandemic preparedness in Central Asia: a policy review of aid volatility, donor dynamics, and health system resilience

**DOI:** 10.3389/fpubh.2026.1865815

**Published:** 2026-07-01

**Authors:** Bakhodir Rakhimov, Jae Wook Choi, Kyung Hee Kim

**Affiliations:** 1 Department of Environmental Hygiene, Tashkent State Medical University, Tashkent, Uzbekistan; 2 Department of Preventive Medicine, College of Medicine, Korea University, Seoul, Republic of Korea; 3 Institute for Environmental Health, Korea University, Seoul, Republic of Korea

**Keywords:** aid volatility, Central Asia, development assistance for health, IHR/SPAR capacity, pandemic preparedness

## Abstract

**Background:**

The COVID-19 pandemic exposed the structural fragility of health systems built on volatile external financing. Central Asia—Kazakhstan, Kyrgyzstan, Tajikistan, Turkmenistan, and Uzbekistan—receives health financing from three divergent donor streams: OECD bilateral and multilateral channels, Chinese Belt and Road Initiative mechanisms, and Eurasian Economic Union frameworks. The 2025 suspension of USAID operations, which eliminated 69–78% of program funding in Kyrgyzstan and Tajikistan, transformed chronic financing instability into an acute system-level shock. The relationship between external health financing volatility and pandemic preparedness in Central Asia has not previously been systematically analyzed.

**Methods:**

This policy and practice review draws on three publicly available datasets: OECD Creditor Reporting System health-sector official development assistance (ODA) (sector 120, all donors, constant 2022 USD, 2010–2023); WHO IHR State Party Self-Assessment Annual Reporting composite scores (2018–2023); and WHO Global Health Expenditure Database Figures (2018–2023). Aid volatility was operationalized as the coefficient of variation of annual health-sector disbursements.

**Results:**

Health sector (ODA) is structurally volatile across all five countries, with coefficient of variation values ranging from 21.3% (Kyrgyzstan) to 53.5% (Uzbekistan). The regional aggregate surged from a pre-COVID-19 mean of USD 160.2 million to USD 307.1 million in 2021, then contracted to USD 227.5 million by 2023. A systematic disconnect between financing volume and preparedness outcomes is evident: Kazakhstan sustained the region’s highest World Health Organization State Party Self-Assessment Annual Reporting (WHO SPAR) scores despite the lowest health aid in absolute terms, while Tajikistan’s current health expenditure of 7.0–8.9% of GDP did not prevent SPAR scores from remaining below the 2024 global average of 64. Kazakhstan’s SPAR score fell from 88 in 2021 to 67 in 2023, coinciding with post-COVID-19 aid contraction—a pattern consistent with financing instability rather than capacity management failure.

**Conclusion:**

Aid volatility, not volume alone, undermines pandemic preparedness in Central Asia. Five policy recommendations are advanced: multi-year pooled financing windows under the World Bank Pandemic Fund; redirecting Chinese Belt and Road Initiative health financing through multilateral channels; expanding the Eurasian Economic Union health security mandate with a dedicated preparedness mechanism; increasing domestic health budgets with ring-fenced IHR capacity lines; and integrating an Aid Volatility Index into the WHO IHR Monitoring and Evaluation Framework. Financing predictability is the primary structural imperative for sustaining preparedness in the post-pandemic era.

## 1 Introduction

The COVID-19 pandemic inflicted an estimated $13.8 trillion in cumulative global economic losses between 2020 and 2024, an outcome that exposed the profound inadequacy of pandemic preparedness systems built, in large part, on volatile and politically contingent external financing (1, 2). In the 2 years immediately following the onset of the pandemic, international Development Assistance for Health (DAH) surged to historic levels: $37.8 billion was disbursed for the health-related Coronavirus disease-2019 (COVID-19) response in 2020 and 2021 alone (3).

Yet this mobilization was exceptional, not structural. By 2023, major Organization for Economic Cooperation and Development (OECD) donors had already begun reducing their official development assistance (ODA) targets: the United Kingdom cut its ODA commitment from 0.7 to 0.3% of gross national income; France reduced its development budget by EUR 742 million; and Germany, Finland, and Switzerland announced further contractions (4). The early dissolution of the United States Agency for International Development (USAID) in 2025 removed approximately $9 billion from the global health financing system in a single year, representing a 21% contraction in total global DAH—the largest single-year decline on record (4, 5). These developments, taken together, underscore what development economists have termed the “panic and neglect” cycle: emergency-driven surges in health financing that dissipate rapidly once the acute threat recedes, leaving recipient health systems exposed to the structural consequences of dependence on unreliable external resources (4).

The damage caused by declining DAH volumes has been extensively documented. Less studied, but equally consequential, is the independent harm caused by the instability—rather than the mere insufficiency—of external financing flows. Aid volatility, defined as year-to-year unpredictability in the volume of external resource transfers, has been shown to undermine fiscal planning, disrupt government expenditure allocations, erode institutional quality, and reduce the macroeconomic effectiveness of aid independently of its aggregate level (6). Lensink and Morrissey demonstrated that the positive impact of aid on economic growth is significantly positive only when the volatility of aid flows is controlled for (6). Hudson and Mosley subsequently showed that both positive and negative aid volatility reduce government expenditure and aggregate investment, with shortfalls in aid having particularly damaging institutional effects (7). More recently, Iannantuoni provided cross-national evidence that aid volatility independently hinders the development of high-quality public institutions in recipient countries, operating through disruptions to resource allocation, tax policy coordination, and civil society engagement (8). Applied to health systems specifically, these mechanisms suggest that volatile external financing undermines precisely those institutional capacities—workforce continuity, supply chain integrity, surveillance system maintenance, and multi-year health planning—that are most essential to pandemic preparedness (9).

Central Asia—comprising Kazakhstan, Kyrgyzstan, Tajikistan, Turkmenistan, and Uzbekistan—represents a geopolitically and analytically distinctive context for examining these dynamics. All five republics emerged from Soviet dissolution in 1991 with inherited Semashko health systems characterized by hospital-centric delivery, centralized procurement, and state-financed workforces that were severely underfunded at independence (10, 11). Over the subsequent three decades, the region received sustained bilateral and multilateral donor engagement from OECD member countries, particularly the United States, Germany, and multilateral organizations including the World Bank and the European Bank for Reconstruction and Development. This engagement was unevenly distributed: from 1991 to 2018, Kyrgyzstan received approximately $8.1 billion in ODA, Tajikistan $5.9 billion, and Uzbekistan $5.8 billion, while Turkmenistan received only $803 million—a distributional asymmetry that reflects both differential aid dependency and the varying political openness of these governments to external partnerships (10, 12).

Alongside Western ODA, the region increasingly attracted Chinese development finance through Belt and Road Initiative (BRI) mechanisms, primarily directed toward infrastructure, energy, and industry rather than health system strengthening (13), as well as regional institutional engagement through the Eurasian Economic Union (EAEU). This multi-donor architecture—characterized by divergent sectoral priorities, political conditionalities, and financing mechanisms—creates a structurally complex financing landscape that has not been systematically analyzed from the perspective of aid volatility and its consequences for pandemic preparedness.

The withdrawal of USAID from the region in 2025 has rendered these structural vulnerabilities acute. According to the Center for Global Development, Tajikistan lost approximately 69% of its USAID-backed programs and Kyrgyzstan 78%, while in Kazakhstan, Turkmenistan, and Uzbekistan, nearly all programs were discontinued (14). Health and education initiatives were among those halted, including programs that had supported health system surveillance, laboratory capacity, and primary care workforce development (10). While China has emerged as a prominent development finance partner in Central Asia, health-sector assistance has thus far represented a comparatively limited component of its overseas financing profile. Consequently, expectations that Chinese financing will substantially offset potential gaps in multilateral health-system funding should be treated with caution (15). These dynamics exemplify what Chitrakar et al. conceptualize as the interplay of chronic volatility—persistent, long-term financial unpredictability that erodes institutional resilience over time—and acute shocks, sudden high-magnitude funding disruptions that trigger collapse in already weakened systems (5, 16). Central Asian health systems, having been exposed to chronic financing instability throughout the post-Soviet transition, now face an acute shock for which their institutional buffers are limited.

Pandemic preparedness in all five Central Asian countries is formally assessed through the World Health Organization International Health Regulations (IHR) monitoring framework, specifically the State Parties Self-Assessment Annual Reporting (SPAR) tool, which evaluates 15 core IHR capacities across 35 indicators on a 0–100 scale (17). Although the predictive validity of SPAR scores for actual pandemic outcomes has been subject to methodological debate—with some analyses finding limited correlation between SPAR scores and COVID-19 mortality (18, 19) and others demonstrating that higher scores were generally associated with lower COVID-19 incidence (20)—the SPAR framework remains the only internationally standardized, annually collected, country-comparable assessment of health system preparedness capacities available for all five Central Asian states. The COVID-19 pandemic both exposed and deepened preparedness gaps in the region: Central Asia experienced a nearly continuous state of outbreak from November 2020 to April 2022, with a second major surge driven by the Omicron variant reaching peak speed exceeding 100 new cases per 100,000 population per week in February 2022 (21). Despite this burden, the region received only $27.2 million through the World Bank Pandemic Fund for a multi-country One Health preparedness initiative—less than 10% of the Fund’s first-round total of approximately $338 million (22, 23). This disproportionately small regional share underscores the structural inadequacy of current multilateral financing for Central Asian preparedness capacity.

Despite the policy urgency of these intersecting dynamics, the relationship between aid volatility and pandemic preparedness in Central Asia has not been the subject of systematic policy analysis. Existing global health financing literature has focused predominantly on Sub-Saharan Africa, where aid dependency is more extensively documented, while Central Asia has received insufficient attention as a distinct geopolitical and health system context (10, 24). No published study has examined the structural implications of the multi-donor financing triangle—OECD bilateral, Chinese BRI, and EAEU mechanisms—for the stability and predictability of health system financing in the region, nor assessed its consequences for IHR preparedness capacity. This gap is analytically important because the region exemplifies a category of countries that are neither the most aid-dependent (as in the case of Tajikistan and Kyrgyzstan) nor the most financially self-reliant (as in the case of Kazakhstan and Turkmenistan), but occupy an intermediate position of structural vulnerability that makes them disproportionately sensitive to both the volume and the predictability of external health financing.

This policy review seeks to address this gap. Drawing on published literature, publicly available donor and health financing databases, and existing policy documentation, it pursues three objectives: first, to characterize the structural patterns of external health financing in Central Asia across the pre-COVID-19 (2010–2019), COVID-19 (2020–2021), and post-COVID-19 (2022–2025) periods, with particular attention to volatility and donor concentration; second, to assess how these financing dynamics interact with IHR/SPAR preparedness capacity across the five countries; and third, to derive evidence-based policy recommendations for both international donors and Central Asian governments aimed at building more stable, diversified, and resilience-oriented health financing systems. By integrating the Aid Volatility/Shocks Framework—which systematically conceptualizes how sudden fluctuations and structural unpredictability in donor funding disrupt domestic fiscal planning—with the empirical literature on aid instability and the political economy of Central Asian health reform, this review contributes to the evidence base needed to prevent the post-pandemic contraction of external health financing from reversing the preparedness gains achieved between 2020 and 2021.

## 2 Methods

### 2.1 Study design

Consistent with the Frontiers in Public Health article type specifications for a policy and practice review (18), this study integrates secondary data analysis to draw on published peer-reviewed literature, publicly available international health financing databases, and grey literature to assess external health financing dynamics in Central Asia and their implications for pandemic preparedness. The methodology combines: (1) a structured narrative literature synthesis; (2) descriptive secondary data analysis using publicly available international databases (OECD CRS, WHO SPAR, WHO GHED); and (3) a structured policy option assessment against pre-defined criteria. This study is not a systematic review or meta-analysis; this distinction is explicitly stated to reflect the nature of the policy review design, which is appropriate for addressing policy questions requiring both empirical data analysis and contextual interpretation—a design commonly employed in global health financing policy research (19, 20). No primary data collection involving human participants was undertaken; ethical approval was not required. All data sources are publicly available and contain no individually identifiable information. The review covers five post-Soviet Central Asian republics—Kazakhstan, Kyrgyzstan, Tajikistan, Turkmenistan, and Uzbekistan—over three analytically distinct periods: pre-COVID-19 (2010–2019), acute pandemic (2020–2021), and post-COVID-19 contraction (2022–2023). The theoretical framework is the Aid Volatility/Shocks Framework of Chitrakar et al. (5), integrated with the established aid volatility literature (6, 7) and applied to the Central Asian context, where no equivalent policy review has previously been published.

### 2.2 Literature search strategy

**Figure 1:**
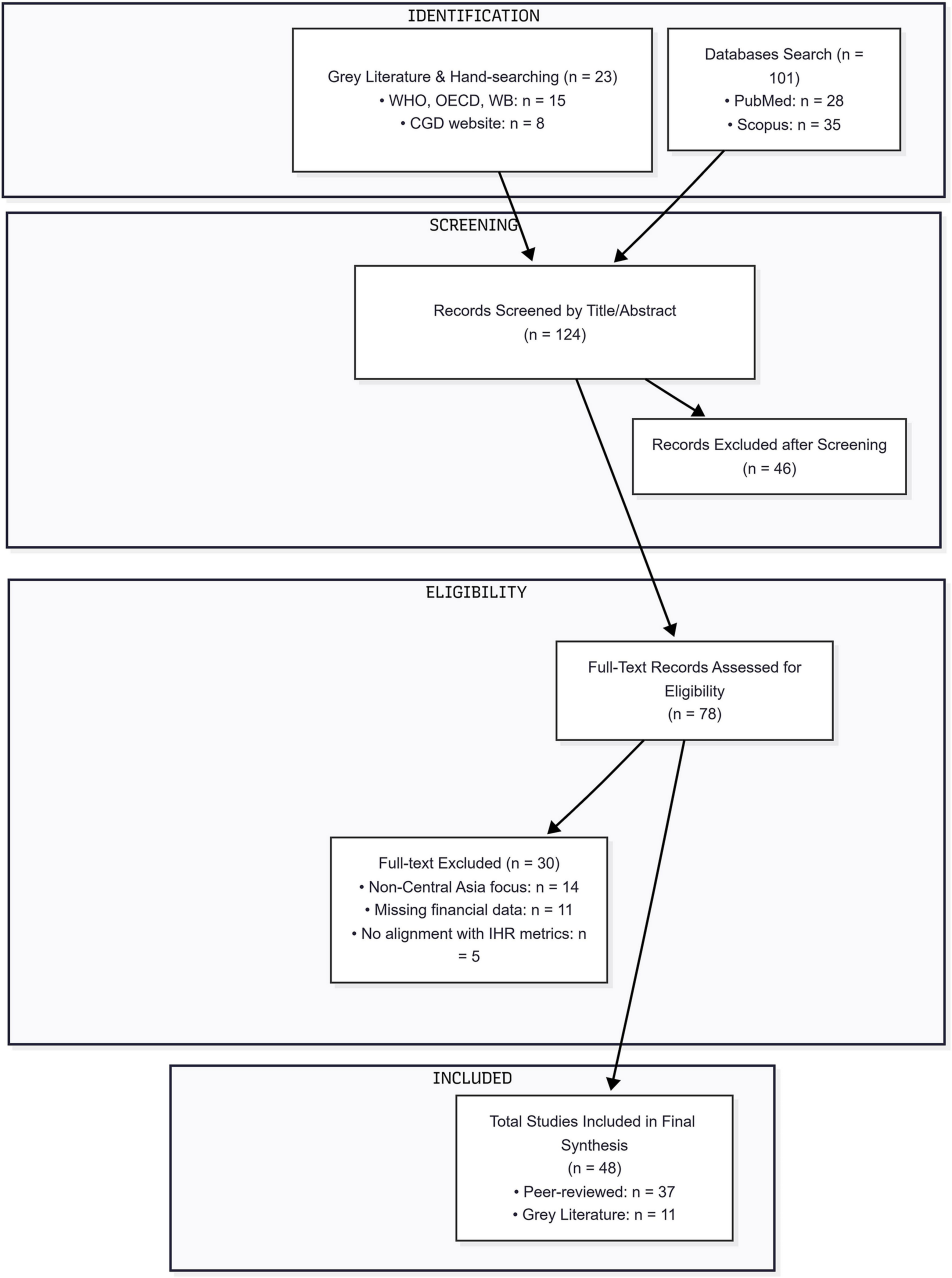
PRISMA flow diagram of the study selection and screening process.

A structured search of peer-reviewed literature was conducted in PubMed, Scopus, and Google Scholar using the following keyword combinations: (“aid volatility” OR “development assistance for health” OR “ODA instability”) AND (“pandemic preparedness” OR “health system resilience” OR “IHR”); (“Central Asia” OR “Kazakhstan” OR “Kyrgyzstan” OR “Tajikistan” OR “Uzbekistan”) AND (“health financing” OR “external aid” OR “donor dependency”); and (“Belt and Road Initiative” OR “EAEU”) AND (“health” OR “preparedness”). To ensure a comprehensive analysis, a targeted literature search was conducted, focusing on English-language publications from January 2000 to April 2026, with priority given to recent dynamics from 2018 onward. Foundational theoretical works on aid volatility published before 2018 were included irrespective of publication date (1, 4, 5). Grey literature was identified through hand-searching the WHO, OECD, World Bank Pandemic Fund, and Center for Global Development websites. Inclusion criteria required substantive engagement with aid volatility, external health financing, or pandemic preparedness as primary topics, published in English between January 2000 and April 2026. Records were screened by title and abstract; duplicates were removed manually. A total of 124 records were identified; of these, 37 peer-reviewed publications and 11 grey literature sources met the inclusion criteria and were retained for the final synthesis. A PRISMA-inspired flow diagram illustrating the selection process is presented in Figure 1. The restriction to English-language literature, which may have excluded relevant Russian-language regional evidence, is acknowledged as a limitation in Section 4.6.

### 2.3 Secondary data sources

Quantitative data on health financing trends were extracted from six publicly accessible databases, summarized in Table 1. The primary financing indicator is health-sector ODA, extracted from the OECD Creditor Reporting System (CRS) for sector code 120 (Health), donor aggregate ALLD (all official donors), flow type disbursements, and constant 2022 USD prices. This indicator captures financing specifically directed at health system strengthening, disease prevention, and health service delivery, in contrast to total ODA which aggregates all development sectors. All monetary values were standardized to constant 2022 United States dollars using the OECD DAC deflator series. Per capita figures were calculated, where noted, using World Bank World Development Indicators (WDI) population data. Pandemic preparedness capacity was assessed using the WHO IHR State Party Self-Assessment Annual Reporting (SPAR) composite scores, which provide annually collected, internationally standardized assessments of 15 IHR core capacities on a 0–100 scale for all five countries (17). The limitations of SPAR as a self-reported measure—including potential upward bias and limited correlation with actual outbreak outcomes—are acknowledged in Section 4.6. The Institute for Health Metrics and Evaluation (IHME) Financing Global Health database was identified as a secondary source for development assistance for health; however, the available dataset extract did not include Central Asian recipients and therefore OECD CRS health-sector data serve as the primary quantitative source for this review.

**Table 1:** Secondary data sources used in this policy review.

Data source	Variables extracted	Coverage	Access
OECD CRS (Creditor Reporting System), Sector 120 Health — data-explorer.oecd.org	Health-sector ODA disbursements; all official donors aggregated (DONOR = ALLD); constant 2022 USD	5 CA countries, 2010–2023	Open, CSV download via OECD Data Explorer
OECD DAC2A (DAC2A: Aid disbursements to countries) — stats.oecd.org	Total ODA (all sectors) for contextual reference; constant 2022 USD	5 CA countries, 2010–2023	Open, CSV download
WHO IHR/SPAR e-platform — extranet.who.int./e-spar	IHR composite scores (0–100), 15 capacities, 35 indicators; SPAR 2nd edition	2018–2024, all 5 countries	Open, country profiles
WHO Global Health Expenditure Database (GHED) — apps.who.int./nha/database	Current health expenditure as % of GDP; government health spending	2010–2023, all 5 countries	Open, interactive + Excel
World Bank World Development Indicators (WDI) — databank.worldbank.org	GDP per capita (NY.GDP.PCAP.KD); population (SP.POP.TOTL); WGI governance indicators	2010–2023, all 5 countries	Open, DataBank export
AidData Global Chinese Development Finance Dataset v3.0 — aiddata.org	Chinese government-financed projects by sector, recipient, year	2000–2021, Central Asia	Open, CSV download
Peer-reviewed literature — PubMed, Scopus, Google Scholar	Aid volatility theory; health financing; IHR/preparedness; Central Asia health systems	English, 2000–2026; priority 2018–2026	Open access and institutional

CA, Central Asia; CRS, Creditor Reporting System; DAH, development assistance for health; GHED, Global Health Expenditure Database; IHR, International Health Regulations; ODA, official development assistance; SPAR, State Party Self-Assessment Annual Reporting; WDI, World Development Indicators.

### 2.4 Analytical approach

The review employs five analytical components, described in Table 2. The Aid Volatility Index (AVI-CV) is operationalized as the coefficient of variation (CV), defined as the standard deviation divided by the mean and expressed as a percentage, of annual health-sector ODA disbursements over the 2010–2023 study period, using data from the OECD CRS. The CV is the standard measure of relative variability used in the aid volatility literature (22, 23) and enables direct comparison across countries with different absolute ODA volumes. The Donor landscape analysis draws on published data and secondary sources for Chinese BRI financing (AidData v3.0) (24, 25) and EAEU-related regional engagement. It acknowledges that data on bilateral health assistance from some regional partners are not consistently available in standardized form for the post-2016 period, representing a recognized limitation discussed in Section 4.6. Policy recommendations in Section 4.5. were assessed against three criteria: feasibility within the Central Asian context, expected impact on health ODA stability, and alignment with existing multilateral frameworks including the WHO IHR Monitoring and Evaluation Framework (26) and the World Bank Pandemic Fund (27, 28).

**Table 2:** Analytical framework: components, data inputs, and outputs.

Component	Description	Primary data	Section
Health ODA trend analysis	Descriptive mapping of health-sector ODA (OECD CRS sector 120) across pre-COVID-19 (2010–2019), COVID-19 (2020–2021), and post-COVID-19 (2022–2023) periods by country and aggregate.	OECD CRS sector 120, GHED.	Section 3.1, Table 4
Aid volatility assessment	Coefficient of variation (CV) computed from annual health ODA disbursements 2010–2023. Comparison with published thresholds from Bulíř and Hamann (22) and Hudson and Mosley (29).	OECD CRS; published aid volatility literature.	Section 3.2, Table 5
Preparedness–financing nexus	Narrative synthesis linking health ODA trajectories to IHR/SPAR composite score trends per country. Country-level paired analysis (ODA vs. SPAR) for 2018–2023.	OECD CRS, WHO SPAR, GHED.	Section 3.3, Table 6
Donor landscape analysis	Comparative assessment of OECD, Chinese (AidData), and EAEU financing streams: sectoral priorities, modalities, and structural contribution to health ODA stability.	OECD CRS, AidData v3.0, published literature.	Section 4.1., 4.2.
Policy option assessment	Structured appraisal of five policy recommendations against feasibility, impact on aid stability, and alignment with existing multilateral frameworks (Pandemic Fund, EAEU, WHO IHR MEF).	Published literature, WHO and World Bank policy documents.	Section 4.5.

AVI-CV, Aid Volatility Index (coefficient of variation); CRS, Creditor Reporting System; EAEU, Eurasian Economic Union; IHR MEF, IHR Monitoring and Evaluation Framework; ODA, official development assistance; SPAR, State Party Self-Assessment Annual Reporting.

The policy option assessment was conducted by the three co-authors independently and consolidated through discussion. Feasibility, expected impact, and timeframe ratings presented in Table 3 represent the authors’ consensus assessment, informed by: (a) existing literature on comparable financing mechanisms; (b) institutional precedent from the Global Fund, Pandemic Fund, and EAEU governance documents; and (c) political economy considerations documented in the Central Asian health systems literature. This assessment does not employ a formal multi-criteria scoring rubric; expert panel validation or Delphi consultation would further strengthen these ratings and is identified as a future research direction in Section 4.7.

**Table 3:** Matrix of health financing implications: stakeholder pathways, institutional feasibility, and systemic horizons.

No.	Recommendation	Lead actors	Feasibility	Timeframe	Expected impact on AVI	Supporting evidence and policy basis
R1	Establish multi-year pooled health financing windows under the Pandemic Fund	G20, World Bank Pandemic Fund, donor governments	Medium	Short–medium (1–3 years)	High	Pandemic Fund 3rd round 2024–2025; G20 HLIP target +$10.5 bln/yr
R2	Redirect Chinese BRI health financing through multilateral channels (WHO, World Bank)	China, WHO, World Bank, SCO health secretariat	Low–medium	Medium (3–5 years)	High	China $783 mln global health aid 2023; BRI sectoral priorities documented by AidData
R3	Expand EAEU health security mandate: dedicated pandemic preparedness financing mechanism	EAEU Eurasian Economic Commission, Kazakhstan, Kyrgyzstan (EAEU members)	Medium	Medium (3–5 years)	Medium	EAEU pharmaceutical regulation harmonization; EAEU Strategy 2025 health provisions
R4	Central Asian governments: increase domestic health budget allocation to ≥5% of GDP	Finance and Health Ministries of Kazakhstan, Uzbekistan, Turkmenistan	Medium	Short–medium (2–4 years)	High	WHO GHED: Kazakhstan CHE 2.87–3.99%; World Bank benchmark $60/capita LIC minimum
R5	WHO IHR MEF: add Aid Volatility Index (AVI) as supplementary SPAR monitoring indicator	WHO IHR Secretariat, States Parties	High	Short (1–2 years)	Medium	2024 IHR amendments; 2025 WHO Pandemic Agreement; IHR MEF framework in force

AVI, Aid Volatility Index (coefficient of variation of health-sector ODA). Feasibility assessed against political, technical, and institutional constraints as of April 2026. Timeframe: short = ≤2 years; short–medium = 1–3 years; medium = 3–5 years. Impact = expected directional effect on health ODA predictability if recommendation is implemented. Feasibility, timeframe, and expected impact are based on the authors’ assessment informed by existing literature and policy precedents.

## 3 Policy assessment

### 3.1 Health-sector ODA trends in Central Asia, 2010–2023

Table 4 presents health-sector official development assistance (ODA) disbursements to each of the five Central Asian states from 2010 to 2023. The five-country regional aggregate reveals a pattern of marked instability punctuated by a sharp COVID-19 driven surge. From a regional total of USD 157.8 million in 2010, health ODA rose moderately across the pre-pandemic decade before accelerating to USD 288.1 million in 2020 and peaking at USD 307.1 million in 2021. This peak represents a 91.1% increase relative to the pre-COVID-19 annual mean of USD 160.2 million (2010–2019).

**Table 4:** Health-sector ODA disbursements to Central Asian states, 2010–2023 (USD millions, constant 2022 prices).

Year	Kazakhstan	Kyrgyzstan	Tajikistan	Turkmenistan	Uzbekistan	5-country total
2010	33.7	32.3	43.6	7.1	41.1	**157.8**
2011	22.7	48.0	32.5	7.3	34.2	**144.7**
2012	18.3	34.7	34.9	3.2	49.7	**140.9**
2013	12.7	35.0	43.6	6.8	36.4	**134.6**
2014	14.0	43.9	45.0	5.7	41.4	**150.0**
2015	7.2	39.5	31.1	4.6	51.5	**133.9**
2016	12.3	33.7	34.3	12.6	81.9	**174.9**
2017	14.5	44.3	50.2	14.1	77.1	**200.3**
2018	10.4	50.5	40.7	3.2	63.7	**168.4**
2019	9.3	40.4	41.4	5.5	100.1	**196.7**
2020	9.5	38.5	55.6	6.5	178.0	**288.1**
2021	13.6	53.8	102.7	10.8	126.2	**307.1**
2022	16.0	65.4	66.0	3.3	77.3	**228.0**
2023	11.7	39.6	76.0	3.4	96.8	**227.5**
Pre-COVID-19 mean (2010–2019)	15.5	40.3	39.7	7.0	57.7	**160.2**
COVID-19 mean (2020–2021)	11.6	46.1	79.1	8.6	152.1	**297.6**
Post-COVID-19 mean (2022–2023)	13.9	52.5	71.0	3.3	87.0	**227.7**

Source: OECD CRS (46). ODA, official development assistance. All values USD millions, constant 2022 prices. Source: OECD CRS, SECTOR = 120 (Health), DONOR = ALLD (all official donors), MEASURE = 100 (ODA disbursements), CHANNEL = _T (all channels). Phase means = arithmetic averages of annual values within each period. Bold values indicate the study-period maximum annual ODA disbursement for each respective country.

Following this transient expansion, regional financing contracted significantly. The five-country aggregate dropped to USD 228.0 million in 2022 and USD 227.5 million in 2023, marking a 25.9% decline from the 2021 peak, though remaining 42.1% above the pre-pandemic baseline.

Country-level trajectories diverge sharply. Kazakhstan—the region’s largest economy and least aid-dependent state—recorded health ODA that declined from USD 33.7 million in 2010 to a study-period minimum of USD 7.2 million in 2015, and remained below USD 17 million throughout the study period (pre-COVID-19 mean: USD 15.5 million). Uzbekistan displayed the most dramatic COVID-19 period surge: health ODA rose from a pre-COVID-19 mean of USD 57.7 million to USD 177.9 million in 2020 (+208% in a single year), before declining to USD 87.0 million in the post-COVID-19 mean—still 50.7% above pre-COVID-19 levels, reflecting sustained multilateral health system reform financing. Tajikistan showed a similar pattern, with health ODA more than doubling from a pre-COVID-19 mean of USD 39.7 million to USD 102.7 million in 2021, before partially contracting in 2022–2023 (post-COVID-19 mean: USD 71.0 million, +78.8% above pre-COVID-19). Turkmenistan experienced the steepest post-COVID decline: health ODA fell from a COVID-19 mean of USD 8.6 million to a post-COVID-19 mean of only USD 3.3 million—a 52.1% reduction below pre-COVID-19 levels—reaching its study-period minimum of USD 3.3 million in 2022.

### 3.2 Aid volatility and the health financing instability gap

Table 5 presents the Aid Volatility Index (AVI), computed as the coefficient of variation (CV) of annual health-sector ODA across the 2010–2023 period. CV values range from 21.3% in Kyrgyzstan to 53.5% in Uzbekistan, with three of the five countries exceeding 39%.

**Table 5:** Health-sector ODA Aid Volatility Index (AVI-CV) and phase comparison by country, 2010–2023.

Country	AVI-CV 2010–2023	Pre-COVID-19 mean (2010–2019) USD m	COVID-19 mean (2020–2021) USD m	Post-COVID-19 mean (2022–2023) USD m	∆ COVID-19 vs Pre	∆ Post vs Pre	Trend
Kazakhstan	44.2%	15.51	11.58	13.87	−25.3%	−10.6%	↓
Kyrgyzstan	20.5%	40.26	46.14	52.48	+14.6%	+30.4%	↑
Tajikistan	38.5%	39.72	79.12	71.01	+99.2%	+78.7%	↑
Turkmenistan	50.6%	7.00	8.63	3.35	+23.4%	−52.2%	↓
Uzbekistan	51.5%	57.73	152.09	87.04	+163.5%	+50.8%	↑

Source: Authors’ calculations from OECD CRS (47). AVI-CV, Aid Volatility Index (coefficient of variation = SD/Mean × 100), computed from OECD CRS sector 120 health ODA, 2010–2023. Pre-COVID-19 = 2010–2019 mean; COVID-19 = 2020–2021 mean; Post-COVID-19 = 2022–2023 mean. ∆ = percentage change relative to pre-COVID-19 mean. ↑ post > pre by >10%; ↓ post < pre by >10%; → within ±10%.

These high CV values reflect structural volatility rather than steady programmatic development. This instability is primarily driven by the extreme fluctuations between the pre-pandemic baseline, the temporary pandemic-era funding surge, and the subsequent post-COVID-19 contraction documented in Section 3.1. This trend confirms that health-sector financing in the region remains highly volatile and unpredictable, as it is disproportionately influenced by emergency-driven and disease-specific bilateral commitments that fluctuate rapidly with shifting donor programmatic priorities.

Kazakhstan’s CV of 45.8% is particularly diagnostic: despite being the least aid-dependent economy in the region, its health ODA exhibited the third-highest volatility, driven by the rapid decline from USD 33.7 million in 2010 to USD 7.2 million in 2015 and subsequent year-to-year fluctuations. Turkmenistan’s CV of 52.5% reflects extreme sensitivity to donor program cycles, with health ODA ranging from USD 3.2 million (2012) to USD 14.1 million (2017). These CV values substantially exceed the threshold identified by Hudson and Mosley as being associated with macroeconomically damaging fiscal instability (7, 29), and represent financing environments fundamentally incompatible with the multi-year workforce planning, supply chain management, and surveillance system maintenance required by the WHO IHR Monitoring and Evaluation Framework (26).

### 3.3 IHR/SPAR preparedness capacity and its health financing context

The paired analysis of regional health ODA trajectories with International Health Regulations State Party Self-Assessment Annual Reporting (IHR/SPAR) scores demonstrates that countries experiencing the most volatile external financing profiles do not exhibit commensurately higher preparedness levels. In several instances, an inverse relationship between funding spikes and functional capacity is observed (Table 6).

**Table 6:** IHR/SPAR composite scores and current health expenditure (CHE) as a percentage of GDP in Central Asian states, 2018–2023.

IHR/SPAR composite score (0–100) — WHO e-SPAR 2nd edition	2018	2019	2020	2021	2022	2023
Kazakhstan	71	82	81	88	80	67
Kyrgyzstan	57	47	52	42	48	48
Tajikistan	58	62	—	57	63	65
Turkmenistan	67	69	68	81	81	81
Uzbekistan	44	57	55	65	70	62

Current health expenditure (% GDP) — WHO GHED	2018	2019	2020	2021	2022	2023
Kazakhstan	2.90	2.87	3.83	3.99	3.75	3.77
Kyrgyzstan	5.01	4.25	4.94	5.35	5.05	4.50
Tajikistan	7.01	6.95	8.89	8.38	7.63	7.44
Turkmenistan	5.55	5.55	5.57	5.49	5.37	5.50
Uzbekistan	4.55	4.84	6.09	6.93	6.62	6.74

Sources: WHO (26, 48). SPAR, State Party Self-Assessment Annual Reporting. Scores 0–100; higher = greater capacity. “—” = no submission recorded. CHE, current health expenditure. GHED, Global Health Expenditure Database.

Kazakhstan illustrates the volatility-preparedness disconnect in the context of strong domestic fiscal capacity. Despite recording the lowest health ODA in absolute terms (pre-COVID-19 mean of USD 15.5 million), it maintained the highest baseline SPAR scores in the region, peaking at 88 in 2021. However, this peak was followed by a 23.9% reduction, with the score dropping to 67 in 2023. This contraction synchronized with the broader regional post-COVID-19 ODA decline to its study-period minimum, while domestic current health expenditure remained relatively stable, transitioning from 3.99% of GDP in 2021 to 3.77% in 2023.

Uzbekistan presents a distinct profile where extreme external financing volatility directly mirrors capacity fluctuations. It experienced the highest health ODA coefficient of variation (CV) in the region (53.5%), with annual disbursements swinging from USD 34.2 million in 2011 to a peak of USD 178.0 million in 2020 before contracting to USD 77.3 million in 2022. Concurrently, its CHE expanded from 4.55% of GDP in 2018 to a peak of 6.93% in 2021. Its SPAR scores running parallel to this trajectory, improving from 44 in 2018 to a peak of 70 in 2022, before declining to 62 in 2023 as external health ODA contracted by 56.6% from its pandemic-era maximum.

Tajikistan combined high aggregate resource expenditure with structural constraints. It recorded the highest current health expenditure as a percentage of GDP in the region, peaking at 8.89% in 2020 and settling at 7.44% in 2023. Concurrently, health ODA peaked at USD 102.7 million in 2021 before stabilizing at a post-COVID-19 mean of USD 71.0 million. Despite these elevated funding streams, Tajikistan’s SPAR capacity scores remained below the regional baseline during the initial years and only moderately recovered to 65 by 2023, indicating that aggregate external resource surges did not translate into immediate, disproportionate gains in reported IHR capacity.

In contrast, Kyrgyzstan’s capacity metrics showed a structural downward trend despite relative stability in domestic financing. While its current health expenditure remained consistent, averaging 4.85% of GDP across the study period, its health ODA dropped significantly from the 2021 peak of USD 53.8 million to USD 39.6 million in 2023. Running parallel to this decline, Kyrgyzstan’s SPAR score decreased from 52 in 2020 to 48 in 2023, exhibiting a continuous degradation that failed to recover post-pandemic.

Turkmenistan displayed a highly decoupled analytical profile. It maintained a nearly static CHE line near 5.50% of GDP throughout the entire 2018–2023 period. Following a mild pandemic-era funding increase (COVID-19 mean: USD 8.6 million), its external health ODA contracted by 52.1% to a post-COVID-19 mean of USD 3.3 million. Despite this sharp external funding contraction, its reported SPAR composite score remained completely unchanged at 81 from 2021 through 2023.

## 4 Discussion

### 4.1 Principal findings in the context of the research questions

This policy review addressed three research questions: (i) how have external health financing flows to Central Asia changed across the pre-COVID-19, COVID-19 peak, and post-COVID-19 periods; (ii) what temporal associations are observable between health-sector ODA volatility and IHR/SPAR preparedness capacity trajectories across the five states; and (iii) what policy options can reduce structural financing instability. The findings from OECD CRS, WHO SPAR, and WHO GHED datasets support four principal conclusions.

First, health-sector ODA to Central Asia is structurally volatile, with coefficient of variation values ranging from 21.3% (Kyrgyzstan) to 53.5% (Uzbekistan) across 2010–2023—levels consistent with or exceeding thresholds identified in the global aid volatility literature as macroeconomically harmful. Bulíř and Hamann documented median CV values of 20–30% for general ODA across LMICs, and our analysis confirms that health-sector ODA is systematically more volatile than general ODA (22). The asymmetric COVID-19-period response—Tajikistan and Uzbekistan received surges of +99.2% and +163.5% above pre-COVID-19 means while Kazakhstan and Kyrgyzstan received less—demonstrates that volatility is structurally determined by donor programmatic priorities and geopolitical alignments extrinsic to recipient health systems’ actual preparedness needs.

Second, the data reveal temporal associations consistent with a volatility-preparedness connection: the causal direction and magnitude of which require inferential analysis. Tajikistan—which received the highest post-COVID-19 ODA mean in the region (USD 71.0 million, 2022–2023) and the highest current health expenditure as % of GDP (7.44–8.89%)—consistently recorded SPAR scores below the 2024 global average of 64%. Conversely, Kazakhstan, despite the region’s lowest health ODA mean (USD 15.5 million pre-COVID-19), sustained the highest SPAR scores through 2021, only declining sharply to 67 in 2023—coinciding with post-COVID-19 ODA contraction to its study-period minimum. This pattern is consistent with Iannantuoni’s finding that aid volatility independently hinders institutional development regardless of aid volume, by disrupting sustained resource allocation, tax policy coordination, and civil society engagement that underlie durable institutional capacity (8). The implication is that the analytic focus of the global health security community must shift from ODA volume maximization to ODA predictability optimization—a reorientation current IHR MEF monitoring instruments do not yet support.

Third, the 2025 USAID withdrawal highlights the operationalization of an acute shock within systems already characterized by chronic instability—a dynamic that aligns with the theoretical framework of Chitrakar et al. and find consistent contextual patterns in our descriptive data (5). Kyrgyzstan and Tajikistan, which experienced a halt in 78 and 69% of USAID-backed programs, respectively, (14), were already exhibiting declining ODA trajectories prior to the withdrawal, while their concurrent SPAR scores were trending downward or remaining stagnant. The withdrawal therefore strikes systems with limited institutional buffers, consistent with the structural dependency dynamics identified by Nonvignon et al. when DAH displaces rather than complements domestic health investment, withdrawal of external financing produces disproportionate system-level disruption (30).

Fourth, the tripartite donor landscape—comprising OECD multilateral channels, Chinese BRI financing, and EAEU mechanisms—may contribute to fragmentation that can, in some contexts, amplify rather than mitigate volatility. Each stream operates through distinct reporting channels, conditionality frameworks, and sectoral priorities, which can complicate coordinated multi-year planning for sustainable IHR capacity development. The documented contraction of the global aid architecture—highlighted by Apeagyei et al. where global DAH contracted from its historical peak of USD 80.3 billion in 2021 to an estimated USD 38.4 billion in 2025 (a 52.2% reduction)—establishes a highly constrained macro-financial context for lower-middle-income countries (4). Within Central Asia, this macro-level downturn runs parallel to the shifting donor priorities and declining bilateral baselines observed in our 2010–2023 regional dataset, reflecting broader systemic vulnerabilities to international financing shocks rather than isolated programmatic adjustments.

### 4.2 Theoretical contributions

This review makes three contributions to the theoretical literature on global health financing. First, it applies the Aid Volatility/Shocks Framework to post-Soviet Central Asia—a region substantially underrepresented in global health financing research, which has historically concentrated on Sub-Saharan Africa. The Central Asian case reveals that the chronic volatility/acute shock distinction retains structural explanatory power even in middle-income contexts where aid dependence is lower in absolute terms. Despite lower baseline dependency, institutional resilience in these states remains structurally constrained by legacy centralized planning frameworks and fragmented post-Soviet infrastructure (11). Rechel et al. previously identified the sustainability and coordination of financing flows—both domestic and external—as the core unresolved challenge for Central Asian health reform (31); our descriptive data demonstrate that this coordination gap persists over a decade later, compounded by systemic post-COVID-19 donor retrenchments.

Second, the country-level data are consistent with a non-linear and potentially temporally lagged association between health ODA and SPAR preparedness scores. Preparedness gains achieved during elevated ODA periods exhibited rapid declines following financing contractions—as observed in Kazakhstan’s composite score shifting from 88 in 2021 to 67 in 2023. While not established through inferential modeling, this pattern aligns with theoretical predictions from the macro-financial volatility literature, which posits that unpredictable aid cycles disrupt institutional capital accumulation and programmatic continuity (23, 32). This trend suggests that preparedness capacity may function as a stock variable that depreciates under financing instability rather than a simple flow variable tracking current ODA levels. This perspective complements Eze et al.’s multi-country analysis of SPAR trends across 186 countries, which documented global IHR capacity improvements during the COVID-19 period without disaggregating financing determinants (17). Our findings indicate that the post-COVID-19 decline in several Central Asian SPAR scores coincides with regional financing instability, which has important implications for how preparedness deterioration is evaluated within international donor accountability frameworks.

Third, this review offers a novel synthesis of the multi-donor financing landscape in Central Asian health by integrating OECD DAC, Chinese BRI, and regional data streams. It suggests that the structural exclusion of non-DAC financing sources from standardized reporting frameworks represents a gap in the global health security monitoring architecture. Fukuda-Parr’s critique of SPAR as an “invisible indicator” that struggles to enter core policy debates despite its relevance to SDG 3d (33) is echoed here: the current IHR MEF exhibits limited capacity to provide actionable early signals of preparedness deterioration because key macro-financial dynamics remain uncaptured by official monitoring frameworks.

### 4.3 The Central Asian health system context: structural constraints and reform legacies

The aid volatility dynamics documented here must be understood against the backdrop of health system structural constraints that pre-date COVID-19. All five states inherited Semashko health systems—centralized, hospital-centric architectures financed through state budgets with limited insurance pooling. Semenova et al. in a comprehensive scoping review of post-Soviet health system evolution across Kazakhstan, Kyrgyzstan, Tajikistan, Turkmenistan, and Uzbekistan, confirm that the Semashko legacy “greatly influenced the organization and governance of healthcare systems, even three decades after the fall of the USSR,” with underinvestment, limited political commitment, and inadequate primary care reform as persistent structural challenges (34). These legacies create specific vulnerability to ODA volatility: in Semashko-derived systems, where workforce salaries, pharmaceutical procurement, and surveillance infrastructure depend on centralized state financing, disruption of external financing flows is transmitted directly to operational capacity rather than absorbed by diversified financing buffers.

The GHED data presented in Table 5 illustrate this most sharply in Tajikistan, where current health expenditure reached 8.89% of GDP in 2020 yet private out-of-pocket payments accounted for the majority of this spending throughout the study period—reflecting the documented failure of public health financing to pool risks adequately (31). This structural distinction—between aggregate health spending and financing channeled toward IHR preparedness functions—underlines the importance of using sector-specific ODA data (OECD CRS sector 120) rather than total health expenditure when assessing the preparedness-financing relationship. The pandemic-induced CHE% GDP spike in 2020 primarily reflects increased household out-of-pocket payments rather than government or donor investment in IHR-relevant preparedness infrastructure, consistent with the pattern documented by De Foo et al. across 15 countries in their analysis of health financing policies during COVID-19 (35).

Kyrgyzstan represents the partial regional exception. Rechel et al. identified it as the only Central Asian country to adopt a sector-wide approach to health financing, achieving the broadest and most sustained reform in the region (31). Semenova et al. and Ulikpan et al. highlight that Kyrgyzstan’s sector-wide approach functioned as a pioneering institutional model for health aid coordination in the region through the 2020s, providing a structured framework for donor alignment despite persistent underinvestment and primary care gaps (10, 34). Yet even Kyrgyzstan’s more effective reform process has not insulated it from health ODA volatility: its CV of 21.3% remains above the global LMIC median, and its SPAR score declined from 52 in 2020 to 42 in 2021 before partial recovery to 48 in 2022–2023. That the region’s most reform-capable health system is not protected from ODA instability consequences reinforces that financing predictability is a structural prerequisite for IHR capacity maintenance, not merely a secondary condition for more advanced systems.

### 4.4 Global health financing in 2025: Central Asia in comparative perspective

These findings acquire additional urgency in the context of the most severe contraction in global DAH since the establishment of the modern health aid architecture. Apeagyei et al. document that global DAH peaked at USD 80.3 billion in 2021, fell to USD 49.6 billion in 2024, and reached an estimated USD 38.4 billion in 2025—levels last seen in 2009—with forecasts projecting stagnation around USD 36 billion through 2030 under current policies (4). For Central Asia, these aggregate trends manifest through direct bilateral program cancelations and indirect contraction of multilateral pools from which the Pandemic Fund, WHO, and World Bank health programs draw.

The systematic review by Chagoma et al. confirms the positive association between sustained DAH and health system capacity while noting that effectiveness is highly context-dependent and diminished when financing is fragmented and unpredictable (36). This directly corroborates our empirical observation of the volatility-preparedness disconnect in Central Asia and supports the argument that addressing ODA instability—through the multi-year pooled financing mechanisms proposed in Section 4.5.—would yield preparedness gains that volume-focused interventions alone cannot achieve. The critical period for intervention is now: the Center for Healthy Development’s 2025 assessment concludes that the uncertain future of development assistance for health financing is expected to lead to considerable decreases in overall funding levels and collaborative efforts over the coming 5–10 years (36), making the window for establishing multi-year pooled commitments before full donor retrenchment narrow but not yet closed.

### 4.5 Implications for policy and practice

The macroeconomic and structural vulnerabilities identified in this study point toward a set of strategic implications for both international donors and domestic health authorities in Central Asia. Rather than isolating financing trends from institutional capacity, the evidence suggests that transforming the regional health security architecture requires shifting from reactive, annual bilateral spending cycles to structurally insulated fiscal mechanisms. The analyzed data generate five distinct pathways for policy integration, conceptualized across international, regional, and national stakeholder groups (Table 3).

#### 4.5.1 R1 establish multi-year pooled health financing windows under the Pandemic Fund for Central Asia

The most structurally impactful intervention available to the international donor community is the replacement of annual bilateral disbursement cycles with multi-year pooled financing commitments that guarantee a floor level of health ODA regardless of geopolitical developments in any single donor country. The World Bank Pandemic Fund—launched in November 2022 and already disbursing a USD 27.2 million multi-country grant to Central Asia and a USD 19 million country-specific grant to Kazakhstan (37)—provides the institutional architecture for such an approach. The Fund’s third call for proposals, approved at USD 500 million in December 2024, demonstrates sustained governance capacity and growing political support: five months into a USD 2 billion short-term fundraising campaign, the Fund secured more than USD 1 billion in new pledges (30, 37). However, demand in the Fund’s first call exceeded supply by a factor of 24, and the total committed resources remain far below the G20 High-Level Independent Panel’s recommended additional investment of USD 10.5 billion per year for pandemic preparedness and response (38).

We recommend that the Pandemic Fund’s Governing Board designate a Central Asia multi-year health financing window with a minimum five-year commitment horizon, aligned with IHR capacity development planning cycles. A rolling five-year commitment mechanism—modeled on the Global Fund’s three-year replenishment cycle, which mobilized USD 6 billion for health systems strengthening in 2024–2026 (28)—would reduce the CV of health ODA in the region by providing a predictable floor that national health ministries could incorporate into multi-year workforce, laboratory, and surveillance planning. The Lusaka Agenda’s call for pooling donor resources and reducing country-level exposure to donor political cycles (12) directly supports this approach. For Kyrgyzstan and Tajikistan, which lost 69 and 78% of USAID programs, respectively (14), such a window would partially compensate for the acute financing shock while addressing the underlying structural instability.

#### 4.5.2 R2 redirect Chinese belt and road initiative health financing through multilateral reporting channels

Chinese development finance represents one of the largest non-OECD sources of external financing in Central Asia, with Belt and Road Initiative (BRI) investments contributing substantially to the region’s infrastructure development and external borrowing landscape (15). However, Chinese bilateral health assistance totalled only USD 783 million globally in 2023 (39) and is systematically directed toward infrastructure, energy, and industry rather than health system strengthening—mirroring the sectoral priorities documented by AidData across BRI recipient countries (25). The absence of China from OECD DAC reporting frameworks creates a structural data gap, limiting coordinated donor planning and preventing Chinese health financing from being systematically captured within the IHR Monitoring and Evaluation Framework (MEF). This asymmetry between the scale of Chinese development finance and its limited allocation to the health sector represents a significant coordination gap in the region’s health financing architecture.

We recommend that the World Health Organization, in collaboration with the Shanghai Cooperation Organization—which includes all five Central Asian countries and China as members or partners—consider developing a voluntary health financing transparency protocol aligned with OECD CRS reporting standards. Drawing on the OECD’s existing engagement with non-DAC providers, including Türkiye and Saudi Arabia, such a protocol could facilitate the more systematic incorporation of China’s health-related contributions to Central Asia into joint donor coordination frameworks.

Simultaneously, the WHO and World Bank could engage with China through the governance structure of the Pandemic Fund—where China participates as a contributing member—to facilitate greater coordination and alignment between BRI-related health-adjacent financing and existing multilateral health financing mechanisms. The emerging provisions of the WHO Pandemic Agreement, including principles of equitable access to health tools and the proposed Coordinating Financial Mechanism (40, 41), provide a normative basis for such coordination.

#### 4.5.3 R3 expand the EAEU health security mandate with a dedicated pandemic preparedness financing mechanism

Three of the five Central Asian countries—Kazakhstan and Kyrgyzstan as member states, and Uzbekistan as an observer—are formally engaged with the Eurasian Economic Union. The Eurasian Economic Commission has established harmonized pharmaceutical regulations, pharmacovigilance standards, and rules governing the circulation of medicines across member states (42). This regulatory harmonization infrastructure may provide an underutilized platform for health security cooperation that could partially buffer the volatility of OECD-sourced health ODA. Tajikistan, while not an EAEU member, participates in other regional cooperation arrangements, which may offer additional avenues for health security dialog.

We suggest that the Eurasian Economic Commission consider expanding the existing EAEU Strategy 2025 health provisions to include a dedicated pandemic preparedness financing component. This could involve exploring options for a regional health emergency reserve, drawing on lessons from existing EAEU financing arrangements in other sectors. Such a mechanism may help provide a counter-cyclical buffer during periods of reduced external health financing, while supporting preparedness capacity across member states. In addition, the EAEU’s unified pharmaceutical procurement framework could be extended or adapted to support coordinated medical countermeasure stockpiling, thereby addressing supply chain preparedness gaps identified in IHR capacity assessments for the region. Implementation would need to be sensitive to member states’ governance preferences and ensure sufficient flexibility and autonomy, reflecting the diverse policy positions within the EAEU.

#### 4.5.4 R4 Central Asian governments: increase domestic health budget allocation and develop ODA shock-absorbing reserves

The data in Tables 4 and 5 confirm that aid volatility, not merely aid volume, drives preparedness deterioration: Tajikistan’s consistently high CHE as % of GDP (7.0–8.9% across 2018–2023) did not prevent SPAR scores from remaining below the global average, while Kazakhstan’s post-COVID-19 SPAR decline from 88 (2021) to 67 (2023) coincided with health ODA falling to its study-period minimum. These patterns suggest that governments must increase domestic health budget allocations not merely to compensate for aid gaps but to insulate core IHR capacity functions—surveillance, laboratory systems, and health workforce—from the annual unpredictability of external financing.

The World Bank’s 2025 Government Resources and Projections for Health (GRPH) report established a minimum benchmark of USD 60 per capita in combined government and donor health spending for low-income countries (43). For Kazakhstan, with its upper-middle income classification and the lowest health ODA dependence in the region, the recommendation is to increase CHE from its current 3.75–3.99% of GDP toward at least 5%, in order to reduce reliance on cyclical ODA and create a domestic buffer sufficient to sustain IHR capacity investments through periods of ODA contraction—rather than framing this as a response to insufficient domestic spending per se. For Uzbekistan, whose CHE of 6.6–6.9% conceals significant out-of-pocket expenditure, the priority is increasing the government share of health spending to protect recurrent IHR-relevant budget lines. For Turkmenistan, its limited ODA uptake reflects documented political constraints on external partnerships rather than domestic fiscal insufficiency; the recommendation is accordingly focused on improving transparency and IHR reporting capacity. For Kyrgyzstan and Tajikistan, where domestic fiscal space is genuinely constrained, the immediate priority is dedicated ring-fencing of IHR capacity budget lines so that pandemic preparedness functions are the last, not the first, to absorb aid shortfalls. Domestic resource mobilization through tobacco and alcohol excise taxes, applied in several LMIC contexts (44), offers an additional mechanism for generating health-specific revenue that is structurally decoupled from OECD donor cycles.

#### 4.5.5 R5 WHO IHR monitoring and evaluation framework: integrate the Aid Volatility Index as a supplementary SPAR indicator

The 2024 amendments to the IHR and the 2025 WHO Pandemic Agreement—adopted by 124 countries at the 78th World Health Assembly—emphasize enhanced transparency and monitoring of pandemic preparedness capacity (40). However, the current IHR MEF primarily assesses preparedness through SPAR and JEE scores, without incorporating measures of the financing stability that underpins these capacities. This creates a potential monitoring gap: countries may maintain relatively stable SPAR scores during periods of increased external health financing, without necessarily strengthening underlying institutional resilience to financing shocks, and may subsequently experience declines in preparedness indicators when external funding contracts. The limited predictive capacity of SPAR for such dynamics—highlighted in previous studies (45) and reflected in the patterns observed here—may partly stem from the absence of financing stability metrics within the monitoring framework.

We recommend that the World Health Organization IHR Secretariat, in consultation with States Parties and the OECD DAC, consider developing and piloting a supplementary Aid Volatility Indicator as part of the IHR Monitoring and Evaluation Framework (MEF)‘s contextual monitoring layer. The indicator could be operationalized as a three-year rolling coefficient of variation (CV) of health-sector ODA (OECD CRS sector 120), calculated annually and reported alongside SPAR scores in the Global Health Observatory. Countries with AVI-CV above a defined threshold—potentially informed by the literature’s commonly cited range of 20–30% as macroeconomically meaningful (22, 23)—could be prioritized for enhanced donor coordination discussions within existing governance platforms, including the Pandemic Fund. This approach appears operationally feasible, given that OECD CRS data are publicly available, regularly updated, and machine-readable, and that the 2024 IHR amendments emphasize strengthened monitoring of financing for preparedness. Implementing this indicator could help transform the IHR MEF from a capacity snapshot system into a more dynamic preparedness–financing monitoring tool, capable of providing early signals of structural vulnerability.

The five recommendations are mutually reinforcing rather than alternative. R1 and R2 address the instability of the external financing architecture; R3 develops a regional counter-cyclical buffer; R4 builds domestic fiscal resilience; and R5 creates the monitoring infrastructure needed to detect and respond to emerging volatility. Together, they constitute a multi-level strategy for transforming the Central Asian health financing environment from one characterized by structural fragility and acute shock vulnerability into one capable of sustaining the IHR preparedness investments that the region’s epidemiological and geopolitical context demands.

### 4.6 Limitations

This review has five principal limitations inherent to its design and the availability of data. First, as a narrative policy and practice review rather than a systematic review or meta-analysis, the literature synthesis is necessarily narrative and selective. Consequently, it cannot establish causal relationships between health ODA volatility and SPAR outcomes with the statistical power of a panel study. For instance, the temporal correlation between Kazakhstan’s post-COVID-19 ODA contraction and its 2023 SPAR decline is consistent with the volatility-preparedness hypothesis but cannot be attributed causally without controlling for concurrent determinants, including governance changes, domestic budget decisions, and institutional shifts.

Second, WHO SPAR scores carry well-documented methodological limitations as self-reported measures (33). Because they are self-reported by member states, they may reflect aspirational or political commitments rather than fully operational capacity levels, particularly in countries without recent Joint External Evaluations (JEE). Fukuda-Parr characterizes SPAR as an ‘invisible indicator’ that struggles to enter policy debates, while Semenova et al. note that public involvement in health policy development was insufficient across all Central Asian states, suggesting that SPAR scores may mask structural gaps (33, 34). This is evident in Turkmenistan’s stable SPAR scores of 81 across 2021–2023 despite a CV of 52.5% and a post-COVID-19 health ODA mean of USD 3.35 million—a resilience difficult to reconcile with its structural constraints without independent JEE data. A similar verification gap applies to Kazakhstan, which has not undergone a recent JEE.

Third, the absence of comprehensive, systematically reported data on non-DAC bilateral financing, regional partnerships, and EAEU-related health assistance represents a significant analytical gap that constrains the donor landscape analysis. While AidData v3.0 (2000–2021) provides valuable insights into Chinese development finance priorities, it does not offer health-sector disaggregation comparable to the OECD CRS. As a result, OECD CRS-based analyses provide only a partial view of the external financing landscape, as some non-DAC development finance flows are not systematically reported within the database. This issue is especially salient in Central Asia, where Chinese development finance and BRI-related investments constitute an important component of the region’s external financing architecture, requiring the use of proxy indicators to approximate their role in health-system development.

Fourth, the temporal boundary of the available data limits the analysis of current geopolitical shifts. The OECD CRS data extend through 2023 and do not capture the full distributional impact of the 2025 USAID withdrawal, whose health-sector consequences will only become quantifiable in future OECD DAC reporting cycles. The policy implications of this review must therefore be understood as applying to a rapidly evolving financing environment whose current trajectory is substantially more adverse than the 2023 data alone suggest.

Fifth, the restriction of the literature search to English-language publications may have excluded relevant Russian-language peer-reviewed and grey literature from Central Asian and Russian academic sources. These regional sources could contain additional empirical evidence on health financing dynamics, ODA utilization, and grass-roots preparedness capacity. This represents a recognized limitation that future syntheses should address through the systematic inclusion of Russian-language databases and regional institutional reports.

### 4.7 Directions for future research

Four research priorities emerge. First, a longitudinal panel analysis using country-year data should test the causal relationship between health-sector ODA volatility and IHR/SPAR scores, controlling for domestic health expenditure, governance indicators, and health system characteristics. Such analysis would build directly on Eze et al. who assessed SPAR trends across 186 countries without examining financing determinants (17), and would provide the causal evidence base that this policy review cannot supply.

Second, a comparable analysis for other geopolitically distinctive aid-recipient regions—the Western Balkans, South Caucasus, and Pacific Island states—where multi-donor triangular architectures similar to Central Asia’s exist would test the generalisability of these findings and contribute to a broader theory of aid volatility effects on health system resilience in middle-income country contexts.

Third, primary qualitative research with national health system officials in Central Asia is needed to characterize how health ministries operationally experience and respond to ODA volatility—through workforce retention decisions, procurement cycles, and surveillance system maintenance—to complement the macro-level quantitative patterns documented here with micro-level implementation evidence.

Fourth, dedicated methodological research on the feasibility and design of an Aid Volatility Indicator as a supplementary IHR MEF monitoring metric—proposed here as Recommendation R5—is warranted, including pilot testing with WHO IHR Secretariat staff and States Parties representatives. Expert panel validation or Delphi consultation on the policy option assessments presented in Table 3 would further strengthen the evidence base for the recommendations advanced in Section 4.5. The findings here provide sufficient empirical motivation to initiate this consultative process.

## 5 Conclusion

This policy review has documented that health-sector ODA to Central Asia is structurally volatile (CV 21–54%), that this volatility is associated with IHR/SPAR preparedness deterioration independently of aggregate ODA volumes, and that the 2025 USAID withdrawal has superimposed an acute financing shock on systems already weakened by chronic instability. These findings challenge the prevailing volume-centric approach to global health financing advocacy and support a reorientation toward predictability as the primary policy objective in the post-pandemic health aid architecture.

The five policy recommendations advanced here—multi-year Pandemic Fund commitments, multilateral channel engagement of Chinese BRI financing, expanded EAEU health security mechanisms, domestic budget increases and shock-absorbing reserves, and integration of an AVI indicator into the IHR MEF—constitute a mutually reinforcing package that addresses financing fragility at its systemic roots. The feasibility and urgency of these measures is supported by the convergence of an enabling normative environment—the 2024 IHR amendments, the 2025 WHO Pandemic Agreement, and the Pandemic Fund’s expanding governance capacity—with a period of financing crisis that makes structural reform more, not less, necessary.

Central Asia has been the subject of intensive donor engagement since Soviet dissolution, yet the health financing architecture that engagement produced is insufficiently stable to sustain the pandemic preparedness investments that the region’s epidemiological position demands. Translating the momentum of the post-pandemic global health security normative agenda into structural financing stability for this overlooked region is both an ethical imperative and a global health security necessity.
